# National epidemiological analysis of the association of COVID-19 vaccination and incidence of COVID-19 cases in Canada, January to August 2021

**DOI:** 10.14745/ccdr.v49i04a07

**Published:** 2023-04-01

**Authors:** Public Health Agency of Canada, COVID-9, Surveillance, Vaccine Coverage, Information System, Vaccine Effectiveness Surveillance, Public Health Risk Science, National Microbiology Laboratory Teams

**Affiliations:** 1 Public Health Agency of Canada, Ottawa, ON

**Keywords:** COVID-19, Canada, epidemiology, surveillance, incidence, cases, vaccination

## Abstract

**Background:**

In December 2020, Canada began its coronavirus disease 2019 (COVID-19) vaccine rollout campaign. Canadians were vaccinated with differing time intervals between doses, vaccine products and vaccine schedules, based on age, timing of vaccination and jurisdiction. The objective of this study is to describe the epidemiology and association between the incidence of COVID-19 cases following vaccination, time since completion of primary series, time between doses and/or product combination and probability of developing severe outcomes.

**Methods:**

The national COVID-19 case data and vaccination coverage data were extracted from the National COVID-19 Surveillance System, and the Canadian COVID-19 Vaccination Coverage Surveillance System. Population estimates from Statistics Canada were used as denominators for rates and for number of people “not fully vaccinated”. Two binomial generalized linear models were constructed for analysis.

**Results:**

Within the analysis period, fully vaccinated (i.e. completed primary series) cases (n=17,206) were more commonly female and older, and had fewer reported severe outcomes relative to not fully vaccinated cases (n=615,999). Episode date of fully vaccinated cases most frequently occurred two months after receiving their second dose, and time-between doses of 29–49 and 50–77 days were most common. The probability of becoming a detected COVID-19 case in not fully vaccinated individuals was higher than those fully vaccinated. Those receiving two doses of AstraZeneca and those with shortest time intervals between doses had higher probabilities of becoming COVID-19 cases.

**Conclusion:**

Findings from Canada’s national surveillance systems support that being fully vaccinated against COVID-19, having a longer time interval between doses and receiving a messenger ribonucleic acid (mRNA) COVID-19 vaccine schedule compared to other vaccines reduce the probability of becoming a case, using data from January to August 2021.

## Introduction

On January 30, 2020, the World Health Organization (WHO) announced the severe acute respiratory syndrome coronavirus 2 (SARS-CoV-2) (coronavirus disease 2019; COVID-19) outbreak as a Public Health Emergency of International Concern, and a pandemic was declared on March 11, 2020. Since then, the pandemic has resulted in significant morbidity, mortality, and threat to the overall well-being of Canadians ((1,2)). Individual and collective actions were heavily relied on, while safe and effective vaccines were under development.

On December 14, 2020, Canada began its COVID-19 vaccine rollout campaign against SARS-CoV-2 infection following the approval of Pfizer-BioNTech Comirnaty, Moderna-Spikevax, AstraZeneca Vaxzevria and Janssen Jcovden (Johnson & Johnson) COVID-19 vaccines ((3)). Due to the anticipated constraints on vaccine supply, initial doses of COVID-19 vaccination were prioritized for key populations. The vaccination campaign began with residents in long-term care facilities, health workers and adults residing in the territories or living in remote and isolated communities. As vaccine supply changed, Canada’s National Advisory Committee on Immunization (NACI) COVID-19 vaccination program’s recommendations evolved. At the time of interest of this article (January 1 to August 31, 2021, i.e., during the Alpha, Gamma and Delta variants’ waves) receiving a second dose of a two-dose vaccine schedule (completing the primary series) was recommended to provide better and longer-term protection against COVID-19 ((4)). The recommended time interval between doses was extended to four months by March 2021, from the initial recommendation of 21 to 28 days; however, intervals differed across jurisdictions based on their vaccination strategies. Vaccine eligibility expanded and, along with recommendations for extending the time interval between doses, all Canadian residents 12 years of age and older were eligible for a first dose by May 2021 ((5–7)). In June 2021, NACI recommended interchangeability of available vaccines and preferential use of messenger ribonucleic acid (mRNA) vaccines to complete primary vaccination series due to the safety concerns that arose with AstraZeneca use (thrombosis with thrombocytopenia) ((6)). As a result, Canadian residents were vaccinated with differing time intervals between doses, vaccine products and vaccine schedules, based on age, timing of vaccination and Province/Territory (P/T) of residence. A timeline of national recommendations is available in **Supplemental material, Figure S1**.

The COVID-19 vaccines have demonstrated high effectiveness against severe outcomes such as hospitalization and death. Assessing the variations in vaccine series and their impact on transmission dynamics within Canada contributes to the growing body of evidence to better define the long-term COVID-19 vaccine performance and future vaccine development policies ((8–18)).

The objective of this article is to describe the epidemiology and explore the association between the incidence of COVID-19 cases following vaccination and time since completion of primary series. The analysis includes a descriptive summary of vaccination coverage and cases following vaccination, and an analysis model of cases following vaccination to investigate whether time since last dose, time between doses, and/or product combination are associated with becoming a COVID-19 case or developing severe outcomes, following adjustments for relevant covariates.

## Methods

### Definitions

Based on NACI recommendations during the period of analysis (January 1 to August 31, 2021), vaccination programs prioritized delivery of primary vaccination series, whereby individuals with a completed primary vaccination series were denoted as “fully vaccinated”. As Canadian vaccination programs evolved in the latter half of 2021, with the rollout of additional and booster doses, the following definitions reflect the vaccine rollout up to August 2021 focusing on primary series completion.

For this analysis, vaccination status of laboratory-confirmed cases was only based on approved vaccines for use by Health Canada for the period of analysis [Pfizer-BioNTech Comirnaty (Pfizer); Moderna Spikevax (Moderna); AstraZeneca Vaxzevria or COVISHIELD (AstraZeneca)] and was defined in the following categories:

• “Fully vaccinated” cases (i.e. case with a complete primary series): episode date 14 days or more after receipt of the second dose in a two-dose series. For this analysis, only those with a complete primary series are included, as the period of analysis predates the Canadian rollout of additional and booster doses.

• “Not fully vaccinated” cases included the following:

o Unvaccinated cases included those who were unvaccinated at the time of their episode date.

o Cases not yet protected from vaccination included those whose episode date occurred less than 14 days after their first vaccine dose.

o Partially vaccinated cases included those whose episode date occurred 14 days or more after their first vaccine dose or less than 14 days after their second vaccine dose.

Month of last dose refers to the month the second dose of a two-dose COVID-19 vaccine was administered. It was used to calculate the number of months since last dose to infection (based on episode date), referred to in this analysis as “months since last dose”.

Episode date was used to temporally classify confirmed cases and refers to symptom onset date. When symptom onset date was unavailable or the case was asymptomatic, episode date refers to either laboratory specimen collection date or laboratory testing date.

### Data sources

Vaccination coverage data were obtained from P/T immunization registries through the Canadian COVID-19 Vaccination Coverage Surveillance System. Data aggregated by 10-year age group, sex, month of last dose, vaccine product received (i.e. Pfizer-Pfizer, Moderna-Moderna, AstraZeneca-AstraZeneca, mixed mRNA, AstraZeneca-mRNA) and time interval between doses, which reflected the varying recommendations on dose intervals throughout the period of interest (see Supplemental material, Figure S1) (0–28 days, 29–49 days, 50–77 days, 78 or more days) were only available for individuals fully vaccinated as of August 14, 2021.

The national COVID-19 case data was extracted from the National COVID-19 Case dataset, which includes detailed case-level information received from all P/Ts and is maintained by the Public Health Agency of Canada (PHAC). For this analysis, COVID-19 case data included basic demographic data, episode dates, severe outcomes, vaccine products received and date of vaccination for each dose administered. Twelve out of 13 P/Ts (excluding Québec) reported case-level vaccination data for the analysis period (January 1, 2021, to August 31, 2021), with data extracted from the COVID-19 case dataset on February 18, 2022.

The July 1, 2021, population estimates provided by Statistics Canada for Provinces and Nunavut, and population estimates provided by the Yukon and Northwest Territories governments, were used as the denominator for the vaccination coverage rate and to calculate the number of people not fully vaccinated ((19)).

### Analysis

Laboratory-confirmed cases 12 years and older, with episode dates between January 1, 2021, and August 31, 2021, were included in the analyses ((20)).

For statistical modelling analyses, cases were aggregated by P/T, age group, sex, vaccination status, and month of episode date. To establish denominators, the coverage data and population estimates were used to determine the number of individuals in each P/T, age group, sex and vaccination status group eligible to become a case each month. Counts of cases were linked to coverage denominators to calculate proportion of individuals that became a COVID-19 case in each aggregate group. Coverage data contained discrete counts of fully vaccinated individuals, meaning vaccination status was classified as either fully vaccinated or not fully vaccinated. Counts of not fully vaccinated individuals were derived by subtracting the number of fully vaccinated from Statistics Canada’s population estimates. For modelling analyses of fully vaccinated individuals, cases were stratified and aggregated by vaccine product series, time interval between doses, and number of months since last dose. In the coverage data, individuals were considered fully vaccinated on the month they completed their primary vaccination series. With respect to number of months since last dose, fully vaccinated individuals were assigned a value of zero months during this month of series completion.

Cases with missing or invalid vaccination date or product, demographic data (age and sex), or episode date were excluded from the analysis (fewer than 0.5% of cases). To align COVID-19 case data with the coverage dataset, cases that received more than two doses of a COVID-19 vaccine, the Janssen COVID-19 vaccine, or a non-Health Canada authorized COVID-19 vaccine were excluded, along with fully vaccinated cases that completed their primary series after August 14, 2021.

### Statistical models

Two binomial generalized linear models were constructed to assess associations between COVID-19 vaccination, vaccination characteristics (i.e. vaccine products received, time interval between doses and time since last dose), and COVID-19 case incidence. In each model, the main response variable was the proportion of individuals that became COVID-19 cases in a month, among their respective cohort. The total number of individuals in each aggregate group was included as weights in the models, and all predictor variables were included as categorical variables. Atlantic Provinces (Newfoundland and Labrador, New Brunswick, Prince Edward Island and Nova Scotia) were grouped as an “Atlantic” jurisdiction, while all Territories were excluded due to low case counts.

A main model (model 1) was fit to data aggregated by jurisdiction, age group, sex, month of episode date and vaccination status. A second model (model 2) was fit to only fully vaccinated groups, aggregated by jurisdiction, age group, sex, month of episode date, vaccine product series, time interval between doses and month of last dose. The variables used to aggregate data were included as predictor variables in each model.

Using both fit models, predicted effects of each level of each variable were calculated. When calculating the effect of a level, all other variable levels were controlled at their average value. For each level, point estimates and 95% confidence intervals were generated for the probability of an individual becoming a detected COVID-19 case. Variable-level *p* values were calculated to assess statistical significance of all predictor variables.

Data cleaning, manipulation, and visualizations were performed in Excel MS Office and SAS V9.4. Statistical analyses were performed in R version 4.0.2.

## Results

**Figure 1:**
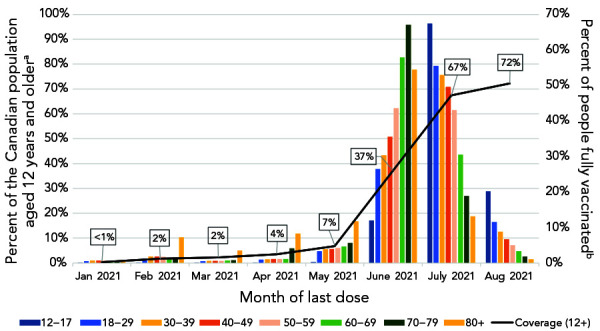
(A) Percent of people fully vaccinated by month of last dose^a,b^ and (B) confirmed cases^c^ in Canada, January to August 2021 ^a^ Percentage of fully vaccinated individuals relative to the 12 years and older population (denominator) ^b^ Percent distribution of fully vaccinated individuals within each age group, by month of last dose (denominator is fully vaccinated individuals within each age group) ^c^ By month of episode and vaccination status

As of August 14, 2021, 72% (n=24,209,666) of individuals 12 years of age and older were fully vaccinated in Canada. Of these, 9% completed their primary series between January and May 2021, and 84% completed their primary series between June and July 2021, given the vaccine rollout in Canada. Among those 50 years of age and older who were fully vaccinated, most completed their series in June 2021 (ranging from 44% in those aged 50–59 years to 67% in those aged 70–79 years). The majority of younger age groups who completed their primary series did so in July 2021 (ranging from 50% in those aged 40–49 years to 67% in those aged 12–17 years) (**Figure 1**).

Across jurisdictions, those in the Territories completed their series earlier than those in the Provinces. Among the Territories, 62% to 75% of those aged 12 years and older completed their primary series by April 2021, compared to 2% to 11% among the Provinces (Supplemental material, **Figure S2**).

There were 633,205 confirmed cases of COVID-19 reported to the national COVID-19 case dataset with episode dates between January 1, 2021, and August 31, 2021, that met analysis inclusion criteria. Of these, 17,206 (2.8%) cases were fully vaccinated at episode date, compared to 615,999 (97.2%) not fully vaccinated at episode date (**Table 1**). See Supplemental material **Figure S3 **for description of case population for analysis. As vaccine campaigns prioritized key populations, including long-term care facility residents, healthcare workers and older adults by decreasing age over time, fully vaccinated cases were older (median age of 45 years compared to 37 years among not fully vaccinated cases) and more commonly female than for the not fully vaccinated cases. The majority of not fully vaccinated cases occurred in April 2021 (n=183,085; 29.7%), while most fully vaccinated cases occurred in August 2021 (n=12,642; 73.5%; Figure 1). There were fewer hospitalizations and deaths reported among fully vaccinated cases compared to not fully vaccinated cases during the period of analysis (Table 1). See Supplemental material **Table S1** for number and percent of those 12 years of age and older who were fully vaccinated by jurisdiction, age group and sex in Canada.

**Table 1:** Descriptive table of national COVID-19 cases in Canada between January 1, 2021, and August 31, 2021

Characteristics	Total (n=633,205)				
	“Not fully vaccinated” cases (n=615,999)		“Fully vaccinated” cases (n=17,206)		Total cases, n
	n	%	n	%	
**Age group (years)**						
12–17	49,733	8.1%	275	1.6%	50,008
18–29	171,734	27.9%	3,533	20.5%	175,267
30–39	121,332	19.7%	3,194	18.6%	124,526
40–49	98,259	16.0%	2,829	16.4%	101,088
50–59	85,091	13.8%	2,504	14.6%	87,595
60–69	50,873	8.3%	2,136	12.4%	53,009
70–79	23,039	3.7%	1,220	7.1%	24,259
80 and older	15,938	2.6%	1,515	8.8%	17,453
**Sex**						
Male	314,935	51.1%	7,185	41.8%	322,120
Female	301,064	48.9%	10,021	58.2%	311,085
**Month of episode**						
January	109,387	17.8%	6	0.0%	109,393
February	54,899	8.9%	87	0.5%	54,986
March	94,244	15.3%	319	1.8%	94,543
April	183,085	29.7%	1,022	5.9%	184,107
May	98,459	16.0%	1,119	6.5%	99,578
June	18,782	3.0%	574	3.3%	19,356
July	11,411	1.8%	1,437	8.4%	12,848
August	45,752	7.4%	12,642	73.5%	58,394
**Province/Territory**						
British Columbia	98,201	15.9%	3,575	20.8%	101,776
Alberta	125,909	20.4%	5,425	31.5%	131,334
Saskatchewan	32,072	5.2%	1,330	7.7%	33,402
Manitoba	26,296	4.3%	607	3.5%	26,903
Ontario	326,645	53.0%	5,923	34.4%	332,568
Atlantic	6,009	1.0%	142	0.8%	6,151
New Brunswick	1,115	0.2%	37	0.2%	1,152
Nova Scotia	3,783	0.6%	72	0.4%	3,855
Newfoundland and Labrador	993	0.2%	25	0.1%	1,018
Prince Edward Island	118	0.0%	8	0.0%	126
Territories	867	0.1%	204	1.2%	1,071
Yukon	441	0.1%	88	0.5%	529
Northwest Territories	213	0.0%	111	0.6%	324
Nunavut	213	0.0%	5	0.0%	218
**Severe outcome**						
Hospitalization	33,523	5.4%	809	4.7%	34,332
Mortality	6,353	1.0%	272	1.6%	6,625
**Months since last dose^a^**						
0 months	N/A	N/A	449	2.6%	449
1 month	N/A	N/A	4,564	26.5%	4,564
2 months	N/A	N/A	7,002	40.7%	7,002
3 months	N/A	N/A	1,994	11.6%	1,994
4 months	N/A	N/A	1,173	6.8%	1,173
5 months	N/A	N/A	623	3.6%	623
6 months	N/A	N/A	1,190	6.9%	1,19
7 months	N/A	N/A	211	1.2%	211
**Time interval between doses^a^**						
0–28 days	N/A	N/A	3,192	18.6%	3,192
29–49 days	N/A	N/A	6,313	36.7%	6,313
50–77 days	N/A	N/A	5,967	34.7%	5,967
78 or more days	N/A	N/A	1,734	10.1%	1,734

Abbreviation: N/A, not applicable

^a^ Data on time since primary series completion, and time interval between doses are only available for fully vaccinated cases

**Figure 2:**
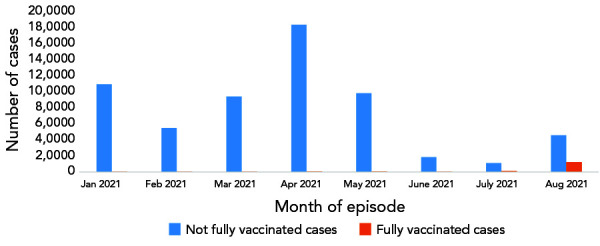
The effect of covariates included in model 1 on predicted probability of an individual becoming a detected COVID-19 case^a,b^ ^a^ Due to large differences in point estimates of variable levels, confidence intervals are narrow relative to y-axis scale. Refer to Supplemental material Table S3 for values of measures of effect and confidence intervals ^b^ Solid points and vertical solid lines show the effect and 95% prediction interval, where visible. Covariates where effects did not differ significantly among levels (only sex) are not shown

The most common vaccine schedule to complete primary series was the Pfizer-Pfizer (63.2%), followed by the Moderna-Moderna (17.5%). Among fully vaccinated cases, the majority received two doses of Pfizer-Pfizer vaccine (n=11,608; 67.5%). Episode date of fully vaccinated cases was most frequent two months after receiving their second dose to complete primary vaccination series (n=7,002; 40.7%), and time-between doses of 29–49 days (n=6,313; 36.7%) and 50–77 days (n=5,967; 34.7%) were most common. See Supplemental material **Table S2 **for number and percent of those 12 years of age and older who were fully vaccinated, by dose interval, month of last dose and vaccine schedule in Canada, as of August 14, 2021. From model 1, variable-level *p* values indicated statistical significance at *p*<0.001 for all predictor variables except sex (*p*=0.18; Supplemental material **Table S3**; see Supplemental material **Figure S4** for predicted effects of sex). Predicted effects demonstrated that the probability of becoming a detected COVID-19 case was higher among not fully vaccinated individuals than fully vaccinated individuals, after adjustment for P/T, age, sex and month of episode date (**Figure 2**). The probability of becoming a detected COVID-19 case of a not fully vaccinated individual was estimated to be 0.204% (95% CI, 0.203–0.206; Supplemental material Table S3). Comparatively, the estimated probability for a fully vaccinated individual was 0.023% (95% CI, 0.023–0.024).

**Figure 3:**
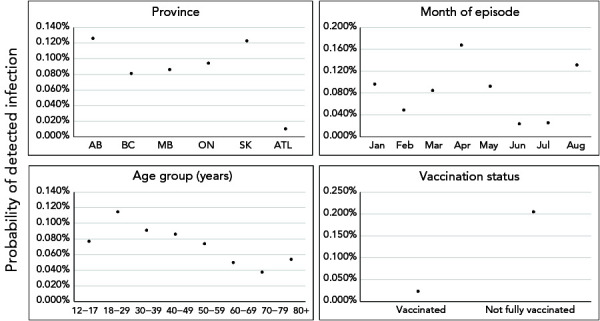
The effect of covariates included in model 2 on predicted probability of “fully vaccinated” individuals becoming a detected COVID-19 case^a^ Abbreviations: AB, Alberta; ATL, Atlantic; AZ-mRNA, AstraZeneca-messenger ribonucleic acid; AZ-AZ, AstraZeneca-AstraZeneca; BC, British Columbia; MB, Manitoba; M-M, Moderna-Moderna; mRNA, messenger ribonucleic acid; ON, Ontario; P-P, PfizerPfizer ^a^ Solid points and vertical solid lines show the effect and 95% prediction interval, where visible. Covariates where effects did not differ significantly among levels (only sex) are not shown. Refer to Supplemental material Table S4 for values of measures of effect and confidence intervals

From model 2, variable-level *p* values indicated statistical significance at *p*<0.001 for all predictor variables except sex (*p*=0.22; Supplemental material **Table S4**). Predicted effects demonstrated that, after controlling for predictor variables, fully vaccinated individuals receiving two doses of AstraZeneca and fully vaccinated individuals with the shortest time interval between doses (0–28 days) had higher probabilities of becoming a detected COVID-19 case, compared to individuals receiving other vaccine series and time interval between doses, respectively (**Figure 3**). Overall, there is a clear signal of increasing probability in becoming a detected case as time since primary series completion increased from zero to six months (Figure 3).

## Discussion

### Key results

At the national level, the results demonstrate that the majority of fully vaccinated 12-year-olds and older completed their series between June and July 2021, with most 50-year-olds and older completing their series in June and those 12–49-year-olds in July. Additionally, cases residing in the Territories completed their series earlier than those residing in Provinces. These observations coincide with the Canada’s vaccine rollout program, which initially targeted the oldest age demographics, high-risk populations and adults residing in the Territories or living in remote and isolated communities. As vaccine eligibility expanded over time with decreasing age, Canadian residents 12 years of age and older were eligible for a first dose by May 2021 ((5)). These results highlighted that the majority of not fully vaccinated cases occurred in April 2021. This is consistent with the timing of the third wave of COVID-19 cases driven by the Alpha variant, as well as the lower vaccination coverage for those 12-year-olds and older (only 4% of this age group was fully vaccinated at the time). The first vaccine dose was being rolled out and eligibility was in the midst of expanding, resulting in lower number of individuals protected against COVID-19 at this time ((21–23)). Of the cases that met analysis criteria, not fully vaccinated individuals had higher case probability compared to fully vaccinated, consistent with international analyses ((15,24,25)). Fully vaccinated cases were more likely to be female and older than not fully vaccinated, as vaccine eligibility initially targeted older populations and people working in healthcare settings—where females are outnumbered ((26,27)).

This analysis further investigated vaccine programmatic factors and whether time since last dose, time interval between doses and/or product combination are associated with becoming a COVID-19 case. Among fully vaccinated individuals, those with a shorter time interval between doses (0–28 days), those receiving two doses of AstraZeneca and those with increased time since last dose from zero to six months, had an elevated probability of becoming a COVID-19 case. Individuals who were vaccinated seven months after their last dose had a lower probability of becoming a case; however, results must be interpreted with caution as the cohort of individuals eligible to become a case seven months after series completion was small (n=211, August 2021), due to the length of the analytic period.

### Comparison

Studies suggest that age ((6,9–14,16,24,25,28–31)), type of vaccine products used ((6,10,12,14,16,24,25,29,31,32)), time interval between doses, time since last dose ((6,9,11–16,18,25,28–34)), vaccination status ((8,12,24,25,29,31)) and predominance of emerging and evolving variants ((9,15,25,31,33)) may impact the duration of protection against COVID-19 and its severity ((6,8–16)). This national analysis is consistent with analyses performed interprovincially and internationally, supporting that vaccine effectiveness against infection decreased across all age groups one to six months after full vaccination ((12)). Results suggest that decreases in vaccine effectiveness may be in part due to waning immunity, as demonstrated by a study conducted in Israel, where those who completed their vaccination series in January and February 2021 were at a 2.26-fold increased risk of developing COVID-19 compared to those who completed their series in March and April 2021 ((18)). A similar trend was observed in a study conducted in England, where greater waning in vaccine effectiveness was observed among older adults, specifically those 65 years and older, and in those who were clinically vulnerable ((6)). Canadian studies conducted in Ontario, British Columbia and Québec highlighted vaccine effectiveness against infection was greater with an mRNA containing schedule compared to two doses of AstraZeneca Vaxzevria vaccine ((31,35)). As for the time interval between doses, studies conducted in the United States and Israel found that time elapsed between the last dose and becoming a case was significantly longer among those with a longer time interval between doses than those with a time interval less than 90 days, with attenuation increasing by month ((28,33)).

### Strengths and limitations

Given Canada’s unique vaccine rollout, this is the first analysis to nationally assess the associations between COVID-19 vaccination status, vaccination characteristics (i.e. vaccine products received, time interval between doses and time since last dose) and case incidence based on these factors. This analysis has high representation across Canada, with 12 of 13 P/Ts providing vaccine information on COVID-19 cases and all P/Ts providing vaccination coverage information; however, variability in reporting across P/Ts may impact interpretation. There are several limitations to acknowledge in this analysis, one of which is that vaccination data were not available for cases from all P/Ts, which may result in an overgeneralization of national results. The expanding vaccine eligibility criteria in Canada over the analysis period (January to August 2021) also presented additional contextual challenges for the interpretation of results, specifically with varying representativeness of Canadian population through analysis period.

Second, this analysis was notably limited by the vaccination coverage dataset providing only counts of fully vaccinated individuals by month of last dose. As a result, only two vaccination status categories could be analyzed—fully vaccinated and not fully vaccinated individuals—as granularity was lost in this latter unconventional classification group. As partial vaccination has demonstrated to reduce the risk of SARS-CoV-2 infection, the inclusion of partially vaccinated individuals in the not fully vaccinated group may impact the estimated effects when comparing by vaccination status ((36–38)). Counts by month of last dose also prevented analyses from precisely accounting for the 14 days to achieve full-vaccination protection in the coverage dataset. Individuals were considered fully vaccinated the month they received their last dose, inflating fully vaccinated coverage estimates for this month (zero months since last dose) and to a lesser extent the following month.

Additionally, this analysis included only cases from the eight-month period following the beginning of vaccine rollout in Canada, limiting the generalizability and size of fully vaccinated coverage groups with longer months since last dose. The limited period of analysis also prevented assessment of severe outcomes, as hospitalizations among fully vaccinated cases were infrequent (n=809) and insufficient for the stratification required for model building.

Lastly, this analysis did not explicitly investigate impacts by public health measures and variants due to limited cases with whole genome sequencing. The period of analysis was performed during the Alpha and Delta waves, which are not explicit effect modifiers on vaccine breakthrough and vaccine effectiveness, as several studies have suggested that vaccine effectiveness and viral burden were reduced with Delta circulation ((6,11,25,30)).

### Interpretation and generalizability

The jurisdictions included in the generalized linear models represent 77% of the Canadian population (9/13 P/Ts) and the jurisdictions included in the descriptive analysis represent 78% of the Canadian population (12/13 P/Ts). Vaccination program rollout and vaccine availability varied across P/Ts and over time; therefore, interpretation at the national level should be done with caution.

## Conclusion

Findings from Canada’s national surveillance systems support that being fully vaccinated against COVID-19, a longer time interval between doses and mRNA COVID-19 vaccines schedule reduce the probability of becoming a COVID-19 case following vaccination. National analyses inform guidance on booster doses and contribute to the growing body of evidence on COVID-19 vaccine performance and vaccine recommendations. Further national analysis on variants, severe outcomes and public health measures may strengthen vaccine effectiveness research and recommendations.

## Supplemental material

These documents can be accessed on the Supplemental material file.
Figure S1: Timeline of relevant vaccine program in Canada for those 12 years of age and older, December 2020 to August 2021
Figure S2: Cumulative percent distribution of “fully vaccinated” individuals by month of last dose and Province/Territory, January 1, 2021 to August 14, 2021
Figure S3: Description of case population for analysis
Table S1: Number and percent of those 12 years of age and older who were “fully vaccinated” by jurisdiction, age group and sex in Canada, as of August 14, 2021
Table S2: Number and percent of those 12 years of age and older who were “fully vaccinated” by dose interval, month of last dose and vaccine schedule in Canada, as of August 14, 2021
Table S3: Predicted effects of variables included in model 1 on probability of becoming a detected COVID-19 case, after controlling for all other variables
Figure S4: Effect of sex on the predicted probability of “fully vaccinated” individuals becoming a detected COVID-19 case in the main model 1, and the model 2 (conditional on full-vaccination status)
Table S4: Predicted effects of variables included in model 2 on probability of becoming a detected COVID-19 case, after controlling for all other variables
